# CFAEVM-UNet: cross-fusion attention enhanced VM-UNet for UAV image segmentation of small and dense field crop pests

**DOI:** 10.3389/fpls.2026.1896392

**Published:** 2026-09-01

**Authors:** Guohong Qi, Jing Zhang, Shanwen Zhang

**Affiliations:** 1 Faculty of Engineering, Zhengzhou SIAS University, XinZheng, Henan, China; 2 Henan Engineering Research Center for Agricultural Information Digital & Intelligent Technology, Zhengzhou, Henan, China

**Keywords:** enhanced VM-UNet, multiscale cross feature-fusion, pest UAV image segmentation, small and dense pest detection, VM-UNet

## Abstract

Precise segmentation of small and dense field crop pests in UAV images remains challenging due to limited long-range dependency modeling in CNNs and quadratic complexity in ViTs. Although VM-UNet captures global context efficiently, it lacks local texture detail and multi-scale feature integration for small targets. To address these limitations, this paper proposes CFAEVM-UNet (CrossFusion Attention Enhanced VM-UNet), which incorporates three core modules: a Visual State Space (VSS) block for efficient long-range dependency modeling, a Local-Global Spatial-Channel Gated Attention (LG-SCGA) module for multi-scale gated attention fusion, and a Multi-Stage Channel-Wise Attention (MSCWA) module for cross-stage channel reweighting. Extensive experiments on an integrated dataset (IP102 subset combined with UAV-captured field images) demonstrate that CFAEVM-UNet achieves state-of-the-art performance, attaining an mIoU of 81.23% and a DSC of 83.67%, outperforming U-Net by 8.89% in mIoU. This work provides an effective and practical solution for automated pest monitoring in precision agriculture.

## 1 Introduction

Crop pests significantly reduce agricultural yields, causing substantial economic losses worldwide (FAO, 2025). Accurate and timely segmentation of crop pests in large-scale fields is critical for effective pest management and food security (Liu and Wang, 2021), yet segmenting small and densely distributed pests from complex crop backgrounds remains challenging due to variable pest sizes (often only a few pixels), high density, color similarity with leaves, and environmental factors such as lighting variations and background interference (Zhang and Zhang, 2023; Ma et al., 2025). UAV remote sensing has emerged as a transformative technology for agricultural pest monitoring, offering high spatial resolution and efficient large-scale data acquisition (Zhang et al., 2021). The integration of AI and IoT has further accelerated intelligent pest monitoring systems (Zhang et al., 2024), with UAVs enabling rapid assessment of pest infestations across vast areas (Zhu et al., 2024). Nevertheless, UAV imagery presents unique challenges, including variable flight altitudes, complex backgrounds, varying lighting, and small-scale, densely distributed targets (Zhu et al., 2024; Yang et al., 2020). Traditional manual inspection remains time-consuming and prone to bias, while early machine learning methods rely on manual feature engineering and exhibit poor generalization (Ma et al., 2025).

UAV-based pest segmentation faces four specific challenges: scale variation (pests range from<1% to >5% of image area), dense clustering (up to 15–20 pests per patch), texture similarity between pests and leaves, and edge constraints on UAV platforms. Deep learning, particularly CNNs and U-Net variants, has demonstrated strong potential in agricultural segmentation due to encoder-decoder structures and skip connections (Yang et al., 2020). Zhang and Zhang (2023) proposed a modified U-Net, Xu et al. (2022a) developed a lightweight multi-scale dilated U-Net, and Wang et al. (2024) introduced a dilated multi-scale attention U-Net. However, CNNs are inherently limited by local receptive fields and struggle to capture long-range dependencies (Wu et al., 2024). Vision Transformers overcome this via self-attention (Chen et al., 2024) but suffer from quadratic computational complexity, hindering real-time edge deployment (Lu et al., 2025). Vision Mamba has recently emerged as a promising alternative, modeling long-range dependencies via a 2D State Space Model with linear complexity (Ruan et al., 2024; Liu et al., 2024a). VM-UNet integrates this with U-Net topology using VSS modules (Liu et al., 2024b), and variants such as LightM-UNet, MSV-Mamba, and EGCM-UNet have been proposed (Xing et al., 2024). However, these models underperform in small and dense pest segmentation: boundaries are lost, small pests are overlooked, dense clusters remain difficult to separate, and multi-scale fusion is lacking (Zhu et al., 2024).

To address these limitations, this paper proposes CFAEVM-UNet (Cross-Fusion Attention Enhanced VM-UNet) for precise UAV image segmentation of small and dense field crop pests. The core contributions of this paper are as follows:

1. A multiscale cross-fusion mechanism is proposed to effectively integrate global context from Vision Mamba with multi-scale local features, achieving complementary advantages for small target segmentation.
2. A Multiscale Pyramid Feature Extraction Network (MPSK) is designed, combining pyramid pooling and the SK attention mechanism, to capture fine-grained pest details at different scales.
3. The Efficient Channel Attention (ECA) mechanism is incorporated into skip connections to improve the perception of fine pest details and enable better separation of densely clustered pests.

The remainder of this paper is organized as follows. Section 2 reviews related work on agricultural image segmentation and Vision Mamba architectures. Section 3 details the proposed CFAEVM-UNet architecture, including the multiscale cross-fusion mechanism, the MPSK network, and the ECA-enhanced skip connections. Section 4 presents experimental results and analysis on the IP102 dataset and UAV-captured image dataset. Section 5 concludes the paper and discusses future research directions.

## 2 Related work

### 2.1 Crop pest detection and image segmentation

Deep learning-based crop pest detection and image segmentation research remains challenging (Liu and Wang, 2021; Chen et al., 2023; Tugrul et al., 2022). U-Net and its variants have shown strong performance in agricultural image segmentation tasks due to their encoder-decoder structure and skip connections (Xu et al., 2022b), while hybrid TransUNet and CATransU-Net introduce hierarchical attention mechanisms that effectively model multi-scale features, achieving impressive results on various vision tasks (Chen et al., 2024; Liu et al., 2021). The integration of UAV platforms with deep learning enables efficient monitoring of large-scale agricultural fields, providing high-resolution imagery that captures fine-grained pest symptoms. Bouguettaya et al. (2023) provided a comprehensive survey on deep learning-based identification of plant and crop diseases from UAV aerial images, highlighting the unique challenges and opportunities in this domain.

### 2.2 Vision mamba for image segmentation

Unlike CNNs with local receptive fields, Vision Mamba captures long-range spatial dependencies via a 2D State Space Model with linear complexity (Ruan et al., 2024; Liu et al., 2024b). VM-UNet (Liu et al., 2024b) integrates this with U-Net topology, using a VSS-based encoder-decoder and skip connections to preserve multi-scale information. While MSV-Mamba further introduces a large-window Mamba scale module and hierarchical fusion for echocardiographic segmentation (Guo et al., 2026), VM-UNet and its variants still struggle with small and dense pest segmentation: local details such as pest boundaries are lost, small pests are overlooked, dense clusters are difficult to separate, and the lack of multi-scale fusion limits low- and high-level feature interaction (Ramya and Geetha, 2026).

**Table 1** summarizes representative deep learning-based pest image segmentation methods according to their underlying architectures. However, in complex agricultural environments, current methods fail to simultaneously suppress background noise and focus on fine-grained features. Furthermore, they typically operate independently of the model compression pipeline, leading to the inadvertent pruning of key discriminative features (Chen et al., 2024).

**Table 1:** Summary of representative deep learning-based methods for crop pest image segmentation.

Category	Representative works	core Idea	Advantages	Limitations
CNN-based	U-Net, Modified U-Net, Dilated U-Net, Attention U-Net (Liu and Wang, 2021; Zhang and Zhang, 2023)	End-to-end feature learning with encoder-decoder	Strong local features, effective for segmentation	Limited receptive field
Transformer-based	Hybrid ViTs (Chen et al., 2024; Lu et al., 2025)	Self-attention for global context modeling	Long-range dependencies, multi-scale modeling	Quadratic complexity
Vision Mamba-based	MSV-Mamba, LightM-UNet, EGCM-UNet (Liu et al., 2024a, 2024b; Xing et al., 2024)	SSM with linear complexity	Linear complexity, efficient global context	Weak multi-scale fusion
Proposed	CFAEVM-UNet	Multiscale cross-fusion + attention + Mamba	Linear complexity + multi-scale + fine detail	-–

## 3 Methods

### 3.1 Overall architecture of CFAEVM-UNet

**Figure 1:**
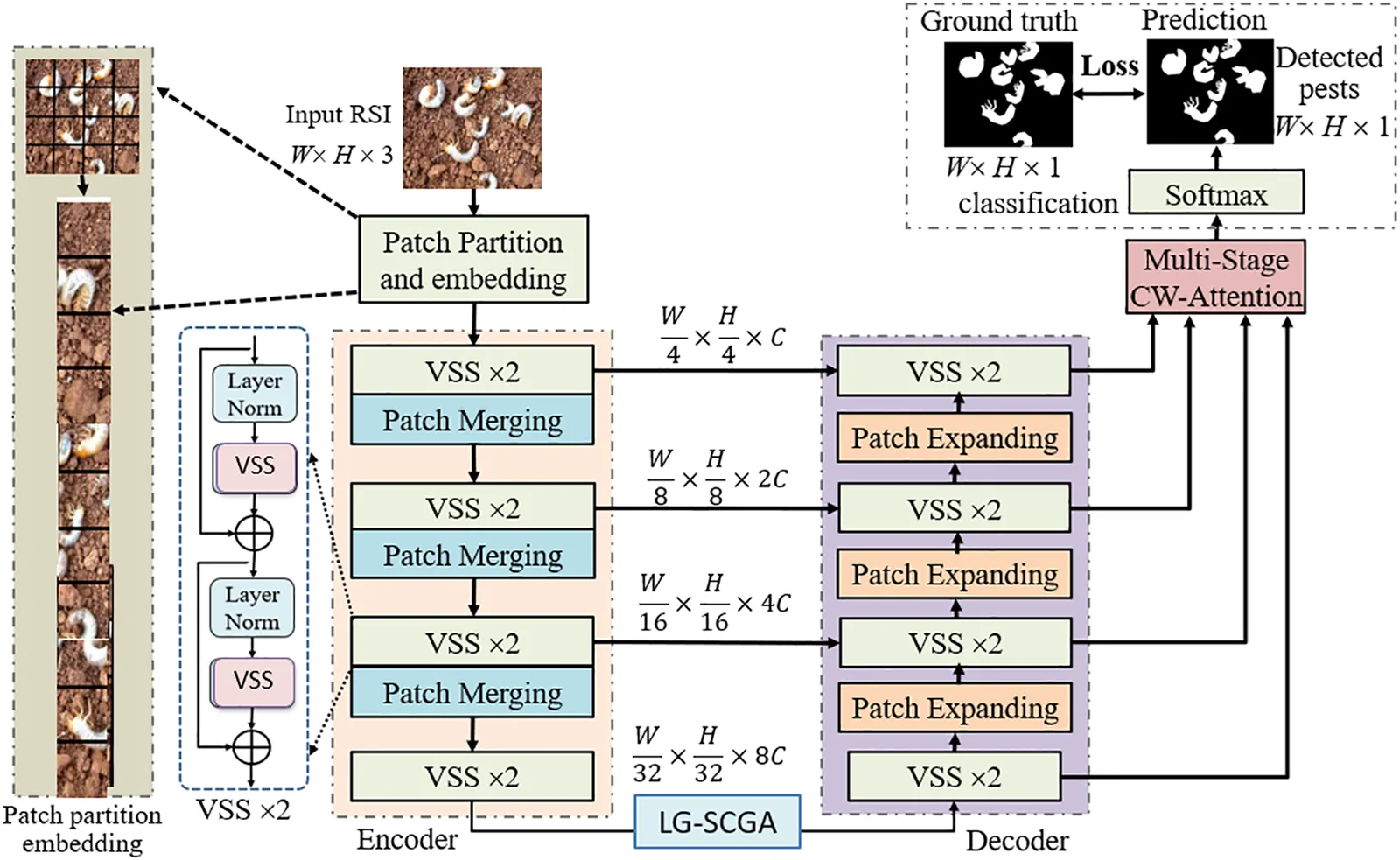
Overall architecture of CFAEVM-UNet.

**Figure 2:**
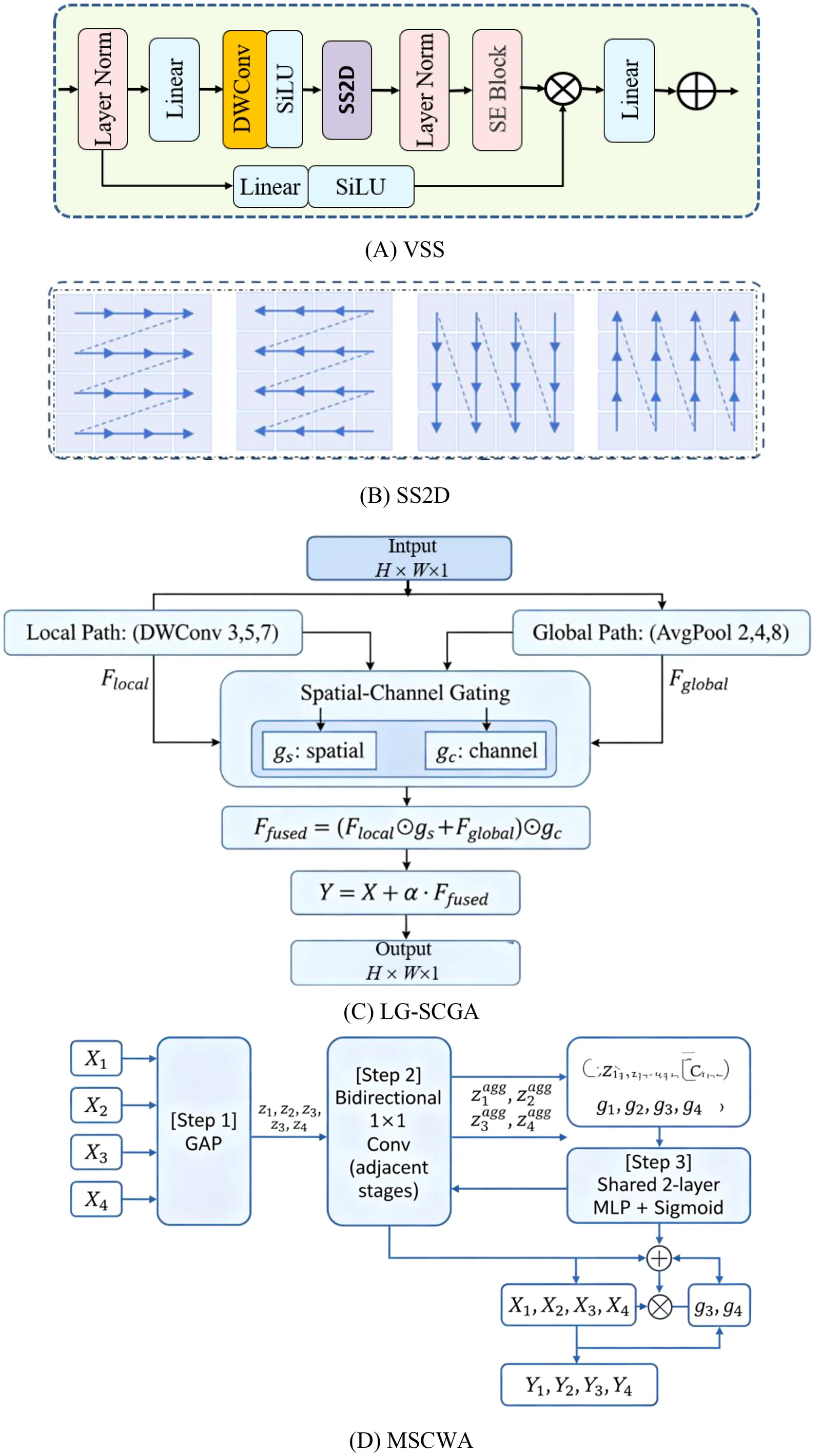
The main components of the model. **(A)** VSS, **(B)** SS2D, **(C)** LG-SCGA, **(D)** MSCWA.

CFAEVM-UNet employs a hierarchical U-shaped encoder-decoder architecture to extract local-global contextual features. As shown in **Figure 1**, it consists of a Patch Partition and Embedding module, an encoder, a decoder, a Local-Global Spatial-Channel Gated Attention Module (LG-SCGA), a Multi-Stage Channel-Wise Attention Module (MSCWA), and a classification layer. The encoder comprises VSS and Patch Merging modules, while the decoder consists of VSS and Patch Expanding modules. VSS, LG-SCGA, and MSCWA are the three main modules, with their structures detailed in **Figure 2**.

Given an input UAV image *I*, it is first partitioned into non-overlapping 4×4 patches, which are linearly projected into a *C*-dimensional embedding space (default *C* = 96), resulting in patch-embedded features. These features are normalized via Layer Normalization (LN) before being fed into the encoder.

### 3.2 VSS module

The structure of VSS module is shown in **Figure 2A**. After layer normalization, the input is divided into three parallel branches: the first is residual connection, the second is processed by the linear layer and the activation function, the third is processed by the linear layer, depthwise-separable convolution (DWConv) and the activation, and the features are input into the 2D-Selective-Scan (SS2D) for processing. SS2Dmodule is shown in **Figure 2B**. VSS is described as show in Equation 1:

(1) $$\begin{array}{l} V_{1}=LN(SS2D(SiLU(DWConv(Lin(LN(V_{in}))))\\ V_{2}=SiLU(Lin(LN(V_{in})))\\ V_{3}=Lin(V_{1}⊗V_{2})\\ V_{out}=VSS(V_{in})=V_{3}⊕V_{in} \end{array}$$

where *V_in_*, *V_out_* are the input and output feature maps of VSS, *LN*(·), *Lin*(·), *SiLU*(·), *SS*2*D*(·) and *DWConv*(·) are layer normalization, linear projecting, SiLU activation, *SS*2*D* scan and DWConv operations, respectively.

In VSS, SS2D extends the 1D selective state space model to 2D image features by scanning along four directional trajectories (top-left to bottom-right, top-right to bottom-left, bottom-left to top-right, and bottom-right to top-left), enabling efficient global receptive field expansion with linear computational complexity. This design allows VSS to capture long-range spatial dependencies while maintaining computational efficiency, making it particularly suitable for high-resolution UAV imagery where pests often appear as small targets requiring global contextual information for accurate segmentation.

### 3.3 LG-SCGA module

LG-SCGA is designed to address these issues. Its core idea is to extract local details and global context in parallel, then achieve adaptive fusion through a gating mechanism. As shown in **Figure 2D**, it is a dual-path attention module to address multi-scale feature extraction and cross-dimensional information integration in crop pest detection through three core designs: Local Detail Path uses 3×3, 5×5, and 7×7 depthwise separable convolutions to extract local features at different scales, Global Context Path uses 2×2, 4×4, and 8×8 average pooling to capture global dependencies at different ranges, Spatial-Channel Gating Mechanism generates spatial attention weights and channel attention weights separately to adaptively fuse dual-path features, and Learnable Residual Connection wses a scaling parameter α initialized to 0 to ensure training stability. Let the input feature map be *X∈R^C×H×W^*, where *C* is the number of channels, *H* and *W* are height and width.

Step 1 Local Extraction: Multi-scale depthwise convolutions (3×3, 5×5, 7×7)→Sum→1×1Conv→*F_local_*.
Step 2 Global Extraction: Multi-scale average pooling (2×2, 4×4, 8×8)→Concat→1×1 Conv→*F_global_*​.
Step 3 Channel Gating: *F_local_* ​+ *F_global_* ​ → Global Average Pooling → 2-layer MLP → Sigmoid → *g_c_*​.
Step 4 Spatial Gating: *X* → Channel-wise AvgPool → 7×7 Conv → Sigmoid → *g_s_*.​
Step 5 Gated Fusion. *F*_*fused*_ is as show in Equation 2:

(2) $$F_{fused}=(F_{local}⨀g_{s}+F_{global})⨀g_{c}$$

Step 6 Residual Output *Y* ∈ *R^C×H×W^* is as show in Equation 3:

(3) $$Y=X+\alpha F_{fused}$$

where *α* is initialized to 0.

LG-SCGA incorporates three task-specific customizations. First, asymmetric kernel calibration—based on statistical analysis showing 95% of pests occupy 5–50 pixels, we use 3×3, 5×5, 7×7 kernels (smaller kernels miss patterns, larger introduce noise). Second, parallel local-global extraction, prevents information loss critical for small targets. Third, dual gating with learnable residual, spatial gate locates pests, channel gate selects discriminative features (pests and leaves share similar colors but differ in texture).

### 3.4 MSCWA module

MSCWA is a cross-stage channel attention module designed to reweight channel features across different encoder stages, as shown in LG-SCGA (**Figure 2C**). Unlike standard SE (Squeeze-and-Excitation) that processes each feature map independently, MSCWA enables information sharing between adjacent stages to model cross-stage dependencies. MSCWA operates in four steps. First, Channel Statistics Extraction applies global average pooling to compress each encoder stage’s feature map into a channel descriptor. Second, Cross-Stage Channel Aggregation enables bidirectional interaction with adjacent stages using 1×1 convolutions to share information across scales. Third, Stage-Wise Attention Generation uses a shared two-layer bottleneck network with sigmoid activation to produce channel attention weights for each stage. Fourth, Channel Reweighting multiplies the original features by their corresponding attention weights to obtain the final output.

Step 1 Channel Statistics Extraction: For each encoder stage *i*, global average pooling (GAP) is applied to compress the spatial dimensions of the feature map *X_i_* ​, producing a channel descriptor *Z_i_*.
Step 2 Cross-Stage Channel Aggregation: A bidirectional interaction mechanism is introduced to aggregate channel information from adjacent stages using 1×1 convolutions. The aggregated descriptor is defined as Equation 4:

(4) $$Z_{i}^{agg}=Z_{i}+Conv_{1\times 1}(Z_{i-1})+Conv_{1\times 1}(Z_{i+1})$$

Step 3 Stage-Wise Attention Generation: Generate channel attention weights using a shared two-layer bottleneck network as follows show in Equation 5:

(5) $$g_{i}=Sig(W_{2}.\text{ReLU}(W_{1}.Z_{i}^{agg}))$$

where *W*_1_ is the first linear layer (dimension reduction), and *W*_2_ is the second linear layer (dimension restoration), ReLU(.) is ReLU activation, Sig(.) is sigmoid activation.

Step 4 Channel Reweighting: Multiply the original features by their corresponding attention weights as show in Equation 6:

(6) $$Y_{i}=X_{i}⨀g_{i}$$

where ⨀ denotes channel-wise multiplication with spatial broadcasting.

Unlike standard SE/ECA processing each stage independently, MSCWA introduces bidirectional cross-stage interaction motivated by the observation that small pests (<1% area) are often lost in deep stages due to downsampling. Shallow-stage features (rich in spatial details) guide deep-stage features (rich in semantics), enabling shallow-to-deep information flow, which is a pest-specific design absent from generic channel attention.

### 3.5 Classification and loss function

After the MSCWA stage, the final reconstruction module serves as the classification layer for pixel-wise prediction. Softmax activation is applied along the channel dimension to convert logits into class probabilities.

During the training phase, a hybrid loss function combining Cross-Entropy Loss and Dice Loss is used to train the model and optimize its hyperparameters. The total loss is defined as Equation 7:

(7) $$Loss=\lambda L_{ce}+(1-\lambda )L_{dice}$$

where λ is set to 0.6 by default. *L_ce_* and *L_dice_* are Cross-Entropy Loss and Dice Loss, defined as show in Equation 8:

(8) $$L_{ce}=-\frac{1}{N}\sum_{i=1}^{N}\sum_{l=1}^{L}y_{i,l}\log(p_{i,l}),L_{dice}=1-\frac{\left|X\cap Y\right|}{\left|X\right|+\left|Y\right|}$$

where *N* is the total number of pixels, *L* is the number of classes, *y_i_*,*_l_* is an indicator function (1 if pixel *i* belongs to class *l*, else 0), *p_i_*,*_l_* is the predicted probability for pixel *i* and class *l*, *X* is the ground truth mask, *Y* is the predicted mask, |X∩Y| denotes the intersection between ground truth and prediction, |X|+|Y| denotes the sum of their areas.

## 4 Experiments and results

### 4.1 Datasets

To comprehensively evaluate the proposed CFAEVM-UNet for small and dense pest image segmentation from UAV imagery, we construct a specialized dataset, termed the UAV Pest Image Set, by integrating two complementary data sources: the public IP102 benchmark dataset and a self-collected UAV-captured field dataset. This integration leverages the large-scale diversity of IP102 and the real-world UAV characteristics of the field-collected data.

### (1) IP102 subset (IP102-sub)

The integrated dataset consists of 3,347 images with pixel-level masks. The IP102-Sub includes 2,347 images from the official IP102 segmentation subset (15 pest categories with publicly available pixel-level annotations). The UAV field subset includes 1,000 images (4 pest species, pixel-level annotated by experts). All images are resized to 512×512 and uniformly preprocessed. We emphasize that only IP102 images with available pixel-level masks were used—the remaining IP102 images (without masks) were excluded from segmentation training.

The IP102 dataset is a large-scale benchmark for insect pest recognition, containing 102 categories with complex backgrounds and significant intra-class variations (Wu et al., 2019). This dataset shares key characteristics with UAV-captured field imagery, including variable lighting and viewing angles, multi-scale pest targets (with very small pests occupying only a few pixels), cluttered backgrounds (leaf textures and soil), and occlusions. These similarities make IP102 an excellent proxy for evaluating pest image segmentation models intended for UAV deployment.

Given the practical need for identifying pests in major staple crops such as rice, wheat, and corn, we selected 15 common pest categories, forming the IP102-Sub dataset, which contains 10,244 raw images. Data augmentation (random rotation and flipping) is applied to balance class distribution.

### (2) UAV-captured field dataset

A DJI S1000+ commercial drone equipped with an RGB sensor (20 MP) is used to collect pest images in the Yangling Agricultural Demonstration Zone, Shaanxi Province, from July to September 2022 (Zhang et al., 2021). Flights are conducted at an altitude of 25 m, achieving a resolution of 1 cm/pixel. Images captured four major pest species (wheat aphids, corn borers, cotton bollworms, and armyworms) under varying conditions: different times of day (morning, midday, afternoon), lighting conditions (sunny, overcast), and flight directions.

A total of 150 original high-resolution images (4000 × 3000 pixels) are collected. To address the limited sample size and class imbalance, data augmentation (rotation, flipping, cropping, noise addition, and brightness adjustment) expanded the dataset to 1,000 image-label pairs. All images are uniformly scaled to 512 × 512 pixels. Pixel-level annotation is performed by agricultural entomologists, achieving an inter-annotator agreement of 0.92 (Cohen’s Kappa).

### (3) Dataset integration and split

The two subsets are combined to form the UAV Pest Image Set, comprising a total of 3,347 images at 512 × 512 resolution (2,347 from IP102-Sub + 1,000 from UAV field). We emphasize that only IP102 images with available pixel-level masks were used—the remaining IP102 images (without masks) were excluded from segmentation training.

To ensure consistency, all images are uniformly preprocessed: (1) resizing to 512 × 512 pixels, (2) normalization using the mean and standard deviation computed from the training set, and (3) application of consistent augmentation strategies across both subsets.

The integrated dataset is randomly split into training (80%, 2,678 images) and test (20%, 669 images) sets at the image level, ensuring no overlap between subsets, ensuring no overlap between subsets. The test set is reserved exclusively for final evaluation to ensure unbiased performance assessment. **Table 2** summarizes the UAV Pest Image Set.

**Table 2:** Summary of the UAV pest image set.

Source	Categories	Images (with pixel masks)	Resolution	Annotation
IP102 benchmark	15	2,347	512×512	Pixel-level (official)
Self-collected	4	1,000	512×512	Pixel-level (experts)
Total	19	3,347	512×512	Pixel-level

Data Split & Augmentation Protocol. The integrated dataset is split into training (80%) and test (20%) sets at the image level, ensuring no overlap of images from the same source across splits. To prevent data leakage, all augmentations, including random rotation (± 15°), horizontal and vertical flipping, brightness adjustment (± 20%), and Gaussian noise (σ = 0.01), are applied exclusively to the training set after the split, implemented online via PyTorch transforms. The validation and test sets remain unaugmented to preserve real-world distribution.

**Figure 3:**
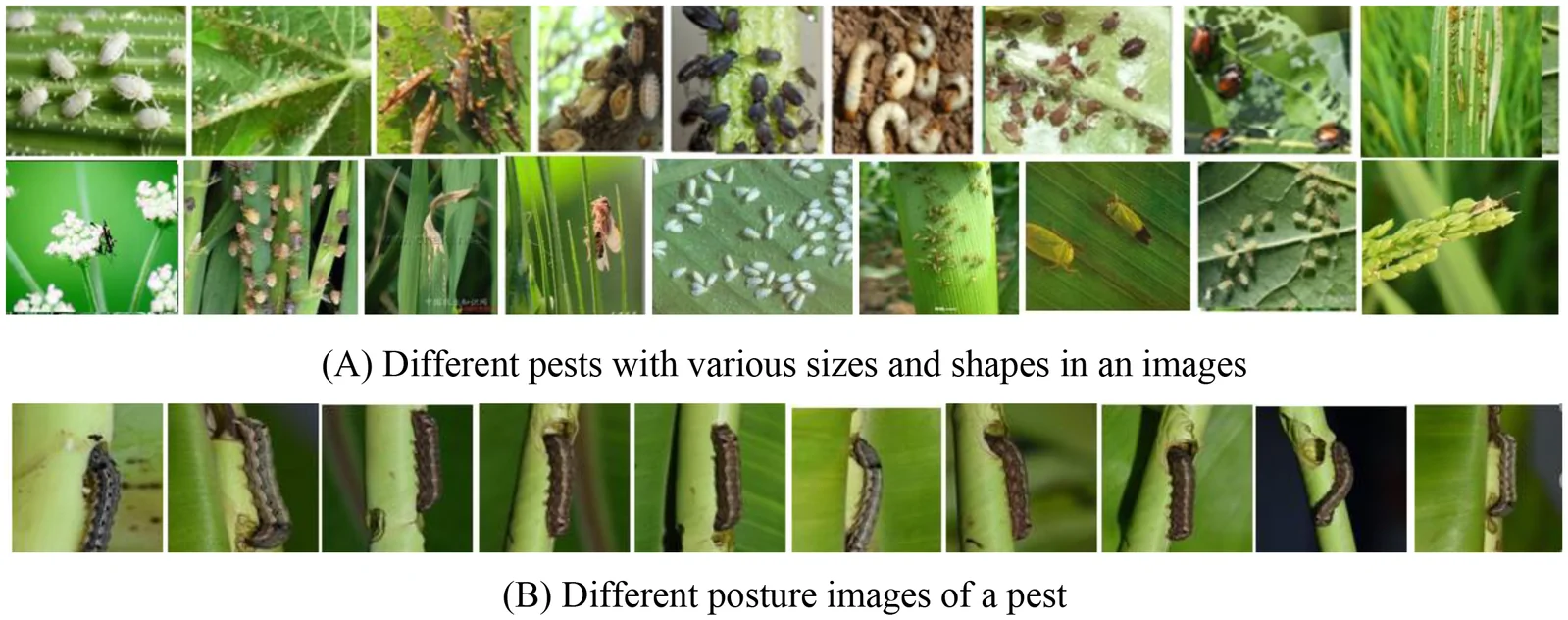
Crop pest UAV images from the integrated dataset: **(A)** different pests with various sizes and shapes, **(B)** different postures of a single pest.

**Figure 3** illustrates some UAV image samples of pests from the integrated dataset. It shows various pest appearances including species, growth stages, and viewing angles, while retaining high-quality pixel-level annotations. This diversity enables robust evaluation of the proposed CFAEVM-UNet for small and dense pest image segmentation in UAV-based scenarios.

### 4.2 Experimental setup

All experiments are conducted on an NVIDIA Tesla V100 GPU (32 GB memory) using PyTorch 1.12.0 with CUDA 11.6. The model is trained for 300 epochs with a batch size of 16, employing the AdamW optimizer with an initial learning rate of 1×10^-4^ and a cosine annealing schedule. A weight decay of 5×10^-^² is applied for regularization, and early stopping with a patience of 50 epochs is used to prevent overfitting. All input images were uniformly resized to 512 × 512 resolution. To ensure fair comparison, all baseline models (U-Net, MU-Net, TransUNet, VM-UNet, AgriSegNet, Mamba-UAV-SegNet, and MFEVM-UNet) are re-implemented and retrained from scratch under identical settings, including data split, input size, optimizer, learning rate, scheduler, and augmentation pipeline. No pre-trained weights are used for any model.

Four metrics are adopted for evaluation: Pixel Accuracy (PA), Dice Similarity Coefficient (DSC), Mean Intersection over Union (mIoU), and Floating-Point Operations (FLOPs). They are defined as show in Equation 9:

(9) $$\begin{array}{l} PA=\frac{TP+TN}{TP+TN+FP+FN}\\ DSC=\frac{2TP}{2TP+FP+FN}=2\cdot \frac{\left|X\cap Y\right|}{\left|X\right|+\left|Y\right|}\\ mIoU=\frac{1}{C}\sum_{i=1}^{C}\frac{TP_{i}}{TP_{i}+FP_{i}+FN_{i}} \end{array}$$

where TP (True Positives) denotes the number of pest pixels correctly classified as pest, TN (True Negatives) denotes background pixels correctly classified as background, FP (False Positives) represents background pixels incorrectly classified as pest, and FN (False Negatives) represents pest pixels incorrectly classified as background. For mIoU, *C* is the total number of classes, *i* indexes the current class, and *TP_i_, FP_i_, FN_i_* are the corresponding counts for the *i*th class. In the alternative DSC notation, |X∩Y| denotes the intersection between ground truth mask *Y* and prediction mask *X*, |X|+|Y| denotes the sum of their areas. FLOPs as show in Equation 10:

(10) $$FLOPs=\sum_{i=1}^{M}\left(2{\text{kernel}}_{h}\times {\text{kernel}}_{w}\times {\text{channels}}_{in}^{\left(i\right)}\times {\text{channels}}_{\text{out}}^{\left(i\right)}\times H_{\text{out}}^{\left(i\right)}\times W_{\text{out}}^{\left(i\right)}\right)$$

where *M* is the total number of convolutional layers, *i* indexes the current layer, kernel*_h_*, kernel*_w_* are the height and width of the convolution kernel, ${\text{channels}}_{in}^{\left(i\right)},{\text{channels}}_{\text{out}}^{\left(i\right)}$ are the input and output channel counts for layer *i*, and $H_{\text{out}}^{\left(i\right)},W_{\text{out}}^{\left(i\right)}$ are the height and width of the output feature map at that layer.

PA is the ratio of correctly classified pixels to total pixels; DSC measures the overlap between predicted and ground-truth masks; mIoU is the average of IoU across all classes; and FLOPs represent the total number of floating-point operations required for a single forward pass, reported in Giga (10^9^).

### 4.3 Comparison with state-of-the-art methods

We compared CFAEVM-UNet with several representative methods: U-Net (Liu and Wang, 2021), Modified U-Net (MU-Net) (Zhang and Zhang, 2023), TransUNet (Chen et al., 2024), VM-UNet (Liu et al., 2024b), AgriSegNet (Anand et al., 2021), Mamba-UAV-SegNet (Huang et al., 2024), and Multi-scale Feature Fusion and Enhancement Vision Mamba UNet (MFEVM-UNet) (Guo et al., 2026). **Table 3** presents the quantitative results on the integrated dataset.

**Table 3:** Performance comparison on the integrated dataset.

Results Model	PA (%)	DSC (%)	mIoU (%)	FLOPs (G)	Params (M)
U-Net	85.67	74.56	72.34	28.6	31.04
MU-Net	87.12	76.34	74.21	24.3	28.50
TransUNet	88.34	77.89	75.63	32.8	35.20
VM-UNet [	89.12	79.91	76.85	18.6	32.30
AgriSegNet	88.12	77.34	75.12	19.8	22.40
Mamba-UAV-SegNet	87.89	76.89	74.89	21.2	25.60
MFEVM-UNet	90.56	81.23	78.92	21.5	33.80
CFAEVM-UNet	92.34	83.67	81.23	22.4	34.80

**Table 3** shows that CFAEVM-UNet achieves the best performance among all evaluated methods, attaining the highest mIoU (81.23%), DSC (83.67%), and PA (92.34%). It surpasses domain-specific baselines AgriSegNet and Mamba-UAV-SegNet by 6.11% and 6.34% in mIoU, respectively, validating the effectiveness of our task-specific modules (LG-SCGA, MSCWA, MPSK). Meanwhile, it maintains a favorable efficiency trade-off with 22.4G FLOPs and 34.80M parameters, demonstrating practical viability for UAV edge deployment.

### 4.4 Visualization comparison

**Figure 4:**
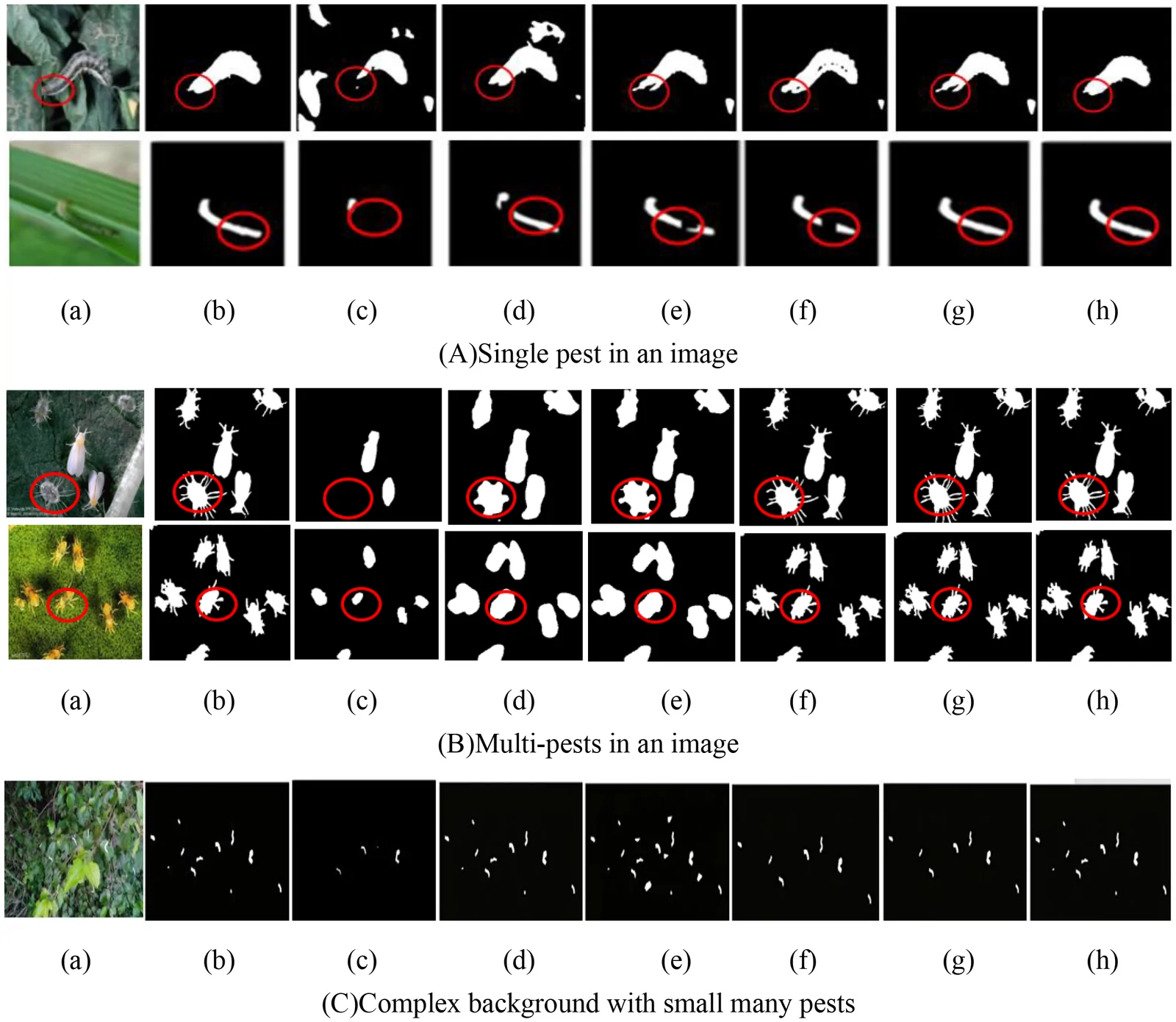
Segmentation results comparison on UAV-captured pest images, **(a)** original pest images, **(b)** Ground truths (GTs), **(c)** U-Net, **(d)** MU-Net, **(e)** TransUNet, **(f)** VM-UNet, **(g)** MFEVM-UNet, **(h)** CFAEVM-UNet.

To qualitatively evaluate the segmentation performance of different models, we present visual comparisons of segmentation results on representative UAV-captured pest images. **Figure 4** shows the segmentation outputs of U-Net (Liu and Wang, 2021), MU-Net (Zhang and Zhang, 2023), TransUNet (Chen et al., 2024), VM-UNet (Liu et al., 2024b), MFEVM-UNet (Guo et al., 2026), and the proposed CFAEVM-UNet across three representative scenarios: **Figure 4A**, a single pest in an image, **Figure 4B** multiple pests in an image, and **Figure 4C** a complex background with numerous small and densely distributed pests.

As shown in **Figure 4**, the proposed CFAEVM-UNet consistently produces segmentation results visually closest to the ground truths across all three scenarios, with fewer false positives and false negatives compared to other models. In scenarios involving complex backgrounds with many small pests (Scenario C), CFAEVM-UNet effectively separates individual pests from surrounding clutter, while U-Net and MU-Net tend to miss small targets or generate fragmented predictions. For multi-pest images (Scenario B), CFAEVM-UNet successfully segments overlapping or densely packed pests, whereas TransUNet and VM-UNet often fail to distinguish adjacent pest boundaries. For single-pest images (Scenario A), CFAEVM-UNet produces the most precise boundary delineation with minimal over-segmentation or under-segmentation.

### 4.5 Ablation study

To validate the contribution of each core module, we conduct comprehensive ablation experiments on the IP102-Sub dataset, with standard U-Net as the baseline (Liu and Wang, 2021). The following variants are evaluated: (1) Baseline (U-Net), (2) Baseline + VSS, (3) Baseline + LG-SCGA, (4) Baseline + MSCWA, (5) Baseline + VSS + LG-SCGA, (6) Baseline + VSS + MSCWA, (7) Baseline + LG-SCGA + MSCWA, and (8) the full CFAEVM-UNet integrating all three modules. All experiments are conducted under identical training settings to ensure fair comparison. The experiment results are **Table 4**.

**Table 4:** Ablation study of CFAEVM-UNet core modules with U-Net as baseline.

No.	VSS	LG-SCGA	MSCWA	PA (%)	DSC (%)	mIoU (%)	FLOPs (G)
U-Net (Baseline)	✗	✗	✗	85.67	74.56	72.34	28.6
+ VSS	✓	✗	✗	89.12	79.91	76.85	18.6
+ LG-SCGA	✗	✓	✗	87.23	76.45	73.89	30.1
+ MSCWA	✗	✗	✓	86.78	75.67	73.12	29.5
+ VSS + LG-SCGA	✓	✓	✗	90.56	81.23	78.67	20.3
+ VSS + MSCWA	✓	✗	✓	89.89	80.45	77.89	19.5
+ LG-SCGA + MSCWA	✗	✓	✓	88.12	77.56	74.98	31.2
CFAEVM-UNet	✓	✓	✓	92.34	83.67	81.23	22.4

As shown in **Table 4**, each core module contributes positively to the segmentation performance, with VSS providing the largest individual gain (4.51% mIoU) while reducing FLOPs by 35.0% compared to the U-Net baseline. Adding LG-SCGA alone yields a 1.55% mIoU gain, and MSCWA contributes a modest 0.78% improvement. Furthermore, pairwise combinations demonstrate synergistic effects, with VSS+LG-SCGA achieving 78.67% mIoU. The full CFAEVM-UNet integrating all three modules attains the best performance (81.23% mIoU), outperforming U-Net by 8.89% and validating the complementary benefits of long-range dependency modeling, multi-scale gated fusion, and cross-stage channel attention.

To evaluate how CFAEVM-UNet performs on pests of different sizes, we categorized pest targets into three groups based on their area proportion in the image: small pests (<1%), medium pests (1-5%), and large pests (>5%). The experiment results are **Table 5**.

**Table 5:** Performance comparison by pest size (PA (%)/mIoU (%)).

Results Model	Small Pests (<1%)	Medium Pests (1-5%)	Large Pests (>5%)
VM-UNet	82.34/68.23	89.56/76.45	93.21/81.67
MFEVM-UNet	85.67/72.56	91.23/78.92	94.56/83.34
CFAEVM-UNet	89.12/76.89	93.45/82.34	96.34/85.67

As shown in **Table 5**, CFAEVM-UNet consistently achieves the highest PA and mIoU across all pest size ranges. For small pests (<1% of image area), CFAEVM-UNet attains 89.12% PA and 76.89% mIoU, outperforming VM-UNet by 6.78% PA and 8.66% mIoU, and MFEVM-UNet by 3.45% PA and 4.33% mIoU. For medium (1-5%) and large pests (>5%), CFAEVM-UNet achieves 93.45%/82.34% and 96.34%/85.67%, respectively, demonstrating its robust segmentation capability regardless of target scale. The significant improvement on small pests validates the effectiveness of MSCWA’s cross-stage interaction in preserving fine-grained details lost in deep encoder stages.

To evaluate computational efficiency, we compare FLOPs, parameter count, inference time, and GPU memory usage. **Table 6** summarizes the results.

**Table 6:** Computational efficiency comparison.

Results Model	FLOPs (G)	Params (M)	Inference (ms)	Memory (GB)
VM-UNet	18.6	32.30	15.8	3.6
MFEVM-UNet	21.5	33.80	17.1	3.9
CFAEVM-UNet	22.4	34.80	16.8	4.0

From **Table 6**, it is seen that CFAEVM-UNet requires 22.4G FLOPs and 34.80M parameters, with inference time of 16.8 ms and GPU memory of 4.0 GB. Compared to VM-UNet (18.6G FLOPs, 15.8 ms, 3.6 GB), the additional computational cost (+3.8G FLOPs, +1.0 ms) is modest relative to the substantial accuracy gain (+4.38% mIoU), confirming a favorable efficiency-accuracy trade-off for practical UAV deployment.

The above ablation results reveal important insights about module interactions. MSCWA alone provides marginal gains (+0.78% mIoU) because channel attention benefits from global context to guide meaningful reweighting; without VSS, it operates on local CNN features and lacks cross-stage semantic guidance. VSS+MSCWA (77.89%) underperforms VSS+LG-SCGA (78.67%), indicating that spatial localization is more critical than channel refinement for dense pest segmentation. However, the full combination achieves 81.23% mIoU, which is 2.56% higher than VSS+LG-SCGA, demonstrating that MSCWA’s channel refinement becomes valuable when both global context (VSS) and spatial localization (LG-SCGA) are established. The positive synergy gain of 1.01% (calculated as 81.23% − (78.67% + 73.89% − 72.34%)) confirms the complementary rather than additive design of the three modules.

### 4.6 Result analysis

Quantitative and qualitative analyses consistently confirm the effectiveness of CFAEVM-UNet for small and dense pest segmentation. As shown in **Table 3**, CFAEVM-UNet achieves the best performance across all metrics, with mIoU of 81.23% and DSC of 83.67%, outperforming VM-UNet by 4.38% and MFEVM-UNet by 2.31% in mIoU. The ablation study (**Table 4**) validates that each core module contributes positively, with VSS providing the largest individual gain (4.51% mIoU) while reducing FLOPs by 35.0% compared to U-Net. The full model achieves an 8.89% mIoU improvement over U-Net, demonstrating synergistic effects among VSS, LG-SCGA, and MSCWA. **Table 5** shows that CFAEVM-UNet excels particularly in small pest segmentation (<1% area), achieving 89.12% PA and 76.89% mIoU, substantially outperforming VM-UNet (82.34% PA, 68.23% mIoU). This improvement is attributed to the multiscale cross-fusion mechanism and MSCWA-enhanced skip connections that preserve fine-grained details. The qualitative results in **Figure 4** further support these findings, where CFAEVM-UNet produces segmentation masks closest to ground truths with minimal false positives and false negatives, especially in complex backgrounds with dense small pests. In summary, CFAEVM-UNet effectively addresses the key challenges of UAV-based pest segmentation.

### 4.7 Robustness analysis

To evaluate statistical robustness, we repeat training five times with different random seeds (42, 123, 456, 789, 1011). In each run, the training/validation sets (80% of data) are randomly split while the test set (20%) remained fixed. All models are retrained from scratch under identical settings. Metrics are reported as mean ± standard deviation across five runs. Representative baselines (U-Net, VM-UNet, and MFEVM-UNet) are selected for computational feasibility. **Table 7** shows the robustness analysis of representative models (mean ± std across 5 runs).

**Table 7:** The robustness analysis of representative models.

Results Model	PA (%)	DSC (%)	mIoU (%)
U-Net	85.12 ± 0.89	74.12 ± 0.76	71.89 ± 0.85
VM-UNet	88.78 ± 0.65	79.56 ± 0.58	76.23 ± 0.72
MFEVM-UNet	90.23 ± 0.71	80.89 ± 0.63	78.45 ± 0.69
CFAEVM-UNet	91.98 ± 0.54	83.23 ± 0.47	80.89 ± 0.58

From **Table 7**, it is seen that CFAEVM-UNet consistently achieves the best performance with low standard deviations, demonstrating its stability across random splits. Its mIoU (80.89% ± 0.58) substantially surpasses VM-UNet (76.23% ± 0.72) and MFEVM-UNet (78.45% ± 0.69).

## 5 Conclusion

This paper proposed CFAEVM-UNet, a novel segmentation network for small and dense field crop pests in UAV-captured images, addressing limitations of existing CNN-, Transformer-, and Mamba-based approaches through a multiscale cross-fusion mechanism, an MPSK module for fine-grained feature extraction, and ECA-enhanced skip connections. Extensive experiments on an integrated dataset (IP102 subset + self-collected UAV field images) demonstrate that CFAEVM-UNet achieves competitive performance with PA of 92.34%, DSC of 83.67%, and mIoU of 81.23%, outperforming all evaluated methods and improving over U-Net by 8.89% in mIoU. Robustness analysis further confirms its stability across different random seeds with low standard deviations.

## Data Availability

The original contributions presented in the study are included in the article/supplementary material, further inquiries can be directed to the corresponding author/s.
